# Workplace stressors and their association with hair cortisol concentrations among ready‐made garment workers in Bangladesh: A cross‐sectional study

**DOI:** 10.1002/1348-9585.12426

**Published:** 2023-09-26

**Authors:** Annegret Dreher, Rita Yusuf, Hasan Ashraf, Syed A. K. Shifat Ahmed, Wei Gao, Christian Strümpell, Adrian Loerbroks

**Affiliations:** ^1^ Faculty of Medicine, Institute of Occupational, Social, and Environmental Medicine, Centre for Health and Society Heinrich‐Heine‐University Düsseldorf Düsseldorf Germany; ^2^ International Center for Biotechnology and Health (ICBH), Center for Health Population and Development (CHPD) Independent University Dhaka Bangladesh; ^3^ Department of Anthropology Jahangirnagar University Dhaka Bangladesh; ^4^ Faculty of Psychology, Chair of Biological Psychology Technical University Dresden Dresden Germany; ^5^ Institute of Social and Cultural Anthropology University of Hamburg Hamburg Germany

**Keywords:** Bangladesh, cortisol, garment industry, hair, work stress, working conditions

## Abstract

**Objectives:**

Psychosocial working conditions of ready‐made garment (RMG) workers have been associated with poorer self‐reported health outcomes. However, no such research has been done with respect to physiological markers that are considered to reflect stress. We consequently aimed to investigate associations of psychosocial working conditions with such a marker, that is, hair cortisol, among RMG workers in Bangladesh.

**Methods:**

We conducted semi‐structured face‐to‐face interviews in labor colonies in the Mirpur area, Dhaka, Bangladesh, in February and March 2021 with individuals identifying as garment workers. The interview inquired after various workplace stressors and resources (i.e., workplace support, workplace bullying, vertical trust, beneficial leadership, work–family conflict, and financial issues including savings, debts, financial obligations, and financial support). In addition, hair samples of 2 cm length were collected from participants. Hair cortisol concentrations (HCC) were determined based on liquid chromatography–tandem mass spectrometry (LC‐MS/MS). Linear regression models were run to detect possible associations of workplace stressors and resources with HCC.

**Results:**

In total, data of 576 participants were included in the analysis (71.9% female, mean age = 25.9 years). Mean HCC was 4.4 pg/mg (standard deviation = 2.1 pg/mg). The sole variable significantly associated with increased HCC was “having to keep your job to support your children or spouse financially” (*β* = 0.28 [95% confidence interval 0.02–0.55]).

**Conclusions:**

The sole workplace stressor significantly associated with increased HCC was the necessity to keep one's job to support children or spouse financially. This observation can, however, barely be disentangled from the fact that one has children/a spouse.

## 1 INTRODUCTION

Bangladesh is among the largest garment exporters worldwide largely supplying European and North‐American markets. With an export value of about 35 billion US dollars in 2021, the transnational garment industry makes up for 82% of the country's total export value.^1^ To date, the industry employs over 4 million ready‐made garment (RMG) workers, a large number of whom are women that work under precarious working conditions such as noise and dust exposure, long working hours, low wages, poor employment security, and frequent workplace harassment and violence.

A previous study by our group examined psychosocial working conditions of RMG workers in Bangladesh.^2^ Those included workplace bullying, workplace support, vertical trust between management and employees, and leadership behavior. We largely found worse psychosocial working conditions to be associated with poorer self‐reported overall health and specific self‐reported health outcomes.^2^ In contrast to self‐reported health measures, however, rarely any studies have analyzed potential associations of workplace stressors and resources with physiological health markers among RMG workers.

Cortisol is a widely used physiological marker of stress. More specifically, in response to a psychosocial stressor, cortisol is secreted by the hypothalamic–pituitary–adrenal axis (HPA‐axis) and can be detected in, for example, saliva, blood, urine, or hair samples.^3^ Hair cortisol concentrations (HCC) are often used to describe chronic stress exposure of individuals as HCC are assumed to reflect long‐term hormone secretion. Compared to cortisol assessment via, for example, blood, saliva, or urine samples, hair sampling is particularly easy to carry out. Only one personal contact is needed for this non‐invasive sampling method. Participants do not need to adhere to complex sampling schemes and samples do not have to be stored in a freezer after collection. Another previous study by our group investigated HCC in a small sample of RMG workers (*n* = 175) and found associations with perceived job promotion prospects.^4^ This study, however, only investigated a limited range of stressors and did not include stressors relevant to RMG workers that have been described in later ethnographic and qualitative literature (e.g., workplace bullying, leadership behavior, work–family conflict, and financial issues). The aim of the present study was, consequently, to investigate the association of workplace stressors and resources with a physiological measure of stress, that is, HCC, more comprehensively and in a larger sample of RMG workers in Bangladesh.

## 2 MATERIALS AND METHODS

### 2.1 Measurements

#### 2.1.1 Development of the interview

The questions for the interview were developed by our multidisciplinary team and piloted among a sample of 56 RMG workers from two garment factories in Uttara and Mirpur, which are both districts of Dhaka (78.6% female, mean age 25.4 years). The interview was shortened from 111 to 74 items after the pilot survey and the original 4‐point response format of most items was simplified to a dichotomous response (yes/no) to enhance comprehensibility.

#### 2.1.2 Interview items

The final interview covered socio‐demographic characteristics (e.g., age, sex, marital status, number of children), health behavior (e.g., smoking), participants' working situation (e.g., duration and type of employment, working hours), and participants' living situation (e.g., safety of one's living place). Eighteen items covered workplace stressors and resources, that is, workplace‐related factors that have been shown to be associated with employee health and factors described as stressful for RMG workers in qualitative and ethnographic research. These were workplace bullying, workplace support, vertical trust between management and employees, leadership behavior, work–family conflict, financial obligations, and financial issues including savings and debt. Participants were asked to specify the presence of each stressor/resource by replying with “yes“/”no“/”not applicable“/”do not want to answer.” Finally, the interview assessed several potential confounders that have been associated with HCC in prior studies, that is, poor self‐reported health,^5^ cardiovascular disease (CVD),^6^ diabetes,^5^, ^6^ and education.

### 2.2 Study sample and procedure

Eligible for participation in the main survey were individuals who were at least 18 years of age and who labeled themselves as RMG workers. We conducted face‐to‐face interviews in labor colonies in one of the oldest garment factory hubs in Dhaka (i.e., Mirpur), Bangladesh, between February and March 2021. This was just a few months after RMG factories had resumed production after months of intermittent lockdowns, cancellation of orders by Western buyers, and mass layoffs of RMG workers. Interviews were conducted by seven female interviewers of a professional survey company who visited colonies in evening hours or on garment workers' days off. Study participants were recruited following a randomized sampling approach: The number of households per colony was estimated and divided by the estimated number of garment workers resulting in a sampling interval (e.g., 3). Interviewers then approached, for example, every third household. In households with more than one garment worker, one worker was randomly selected for participation. All participants provided written informed consent. If participants were younger than 18 years, the interview was immediately terminated, and these participants were excluded from the study. The study was approved by the Bangladesh Medical Research Council (BMRC, Reference BMRC/NREC/2019–2022/705) and the ethics committee of the Medical Faculty of the Heinrich‐Heine‐Universität Düsseldorf, Germany (study number 5427).

### 2.3 Hair sample collection and analysis of hair cortisol

Participants were asked to provide two hair strands of at least 2 cm length and 3 mm width. Samples were taken as close as possible to the scalp approximately 2 cm below the cranial bone. Assuming hair growth of 1 cm per month, the collected samples reflected hair growth of the last 2 months. Sampling procedure and segment length were identical to our previous study among Bangladeshi RMG workers.^4^ Hair cortisol concentrations were determined from liquid chromatography–tandem mass spectrometry (LC‐MS/MS, intra‐ and inter‐assay CVs between 3.7% and 8.8%^7^) performed at the laboratory of the Department of Biopsychology, TU Dresden.

### 2.4 Statistical analysis

For statistical analyses, only RMG workers who stated to have mainly worked in the RMG sector in the past 3 months were eligible (i.e., post‐hoc criterion). Descriptive analysis of the socio‐demographic characteristics of the study population and of workplace stressors and resources was done by displaying absolute numbers and percentages. Cortisol data were reported as mean with respective standard deviation. Z‐scores were calculated for HCC and outliers with standardized residuals exceeding 3.0 were excluded (in keeping with our prior study).^4^ Cortisol data were then ln‐transformed to approximate a normal distribution. Linear regression models were run using the in‐transformed HCC as outcome and workplace stressors and resources as independent variables (each variable in a separate model). The variable “number of people depending on a worker's wage” was included as a continuous variable, all other stressors were included as categorical variables (see Table A1). The exact amounts of monetary savings and debt were dichotomized into variables “any savings” and “any debt,” respectively. Additionally, the amounts of savings and debt were compared to workers' individual income referring to savings/debt as much as “less than 1 monthly salary,” “1–2 monthly salaries,” and “3 or more monthly salaries.” Firstly, models were adjusted for only age and sex. Secondly, models were adjusted for poor self‐reported health, CVD, diabetes, and education. The original 5‐point Likert scale of self‐reported health was dichotomized into good health (very good/good) versus poor health (moderate/bad/very bad). Regression analyses were run using IBM SPSS 27, and results are displayed as *β* coefficients with respective 95% confidence intervals.

## 3 RESULTS

We visited 4375 households and garment workers lived in 1827 of them (41.8%). In total, 1264 workers completed the interview (69.2%) and 659 provided hair samples (36.1%). Interview data and hair samples were available for 649 workers. After exclusion of workers who stated not to mainly have worked in the RMG sector in the last 3 months (post‐hoc inclusion criterion), data of 576 workers were included in the analyses. Socio‐demographic characteristics of the study participants are displayed in Table 1. The study sample was predominantly female (71.9%), young (mean age 25.9 years, standard deviation 7.1 years), married (63.9%), and less educated (61.7% had no education or up to grade 5).

**TABLE 1:** Socio‐demographic and work‐related characteristics of *n* = 576 ready‐made garment workers.

Participant characteristics	Total *n* (%)
Categorical variables	
Sex	
Female	414 (71.9)
Male	162 (28.1)
Highest level of education	
No formal education	88 (15.3)
Grade 1–5	267 (46.4)
Grade 6–10	185 (32.1)
Lower secondary exam (matric/SSC)	24 (4.2)
Higher secondary exam (Intermediate/HSC)	11 (1.9)
Bachelor's degree	1 (0.2)
Postgraduate degree	0 (0.00)
Marital status	
Married	368 (63.9)
Separated or divorced	36 (6.3)
Never married	158 (27.4)
Husband/wife died	14 (2.4)
Currently smoking tobacco products	
Yes	136 (23.6)
No	440 (76.4)
Self‐reported health	
Very good	84 (14.6)
Good	229 (39.8)
Moderate	203 (35.2)
Bad	46 (8.0)
Very bad	14 (2.4)
Diagnosed cardiovascular disease	
Yes	17 (3.0)
No	559 (97.0)
Diagnosed diabetes	
Yes	8 (1.4)
No	568 (95.7)
Bullied by colleagues	98 (17.0)
Bullied by supervisors	84 (14.6)
Trust in information from the management	464 (80.6)
Perceives management to trust workers	531 (92.2)
Supervisors do not care about workers' problems	253 (44.1)
Supervisors take decisions free of personal bias	423 (73.4)
Support from colleagues	443 (76.9)
Support from supervisors	474 (82.3)
Family life has disturbed job	93 (16.1)
Faced problems in family due to job	75 (13.0)
Needs to keep job to financially support spouse or children (for *n* = 422 workers with spouse/children)	405 (96.0)
Needs to keep job to financially support other relatives	353 (61.3)
Could call on someone for financial help	196 (34.0)
Reports to have any savings	142 (24.7)
Reports to have any debt	243 (42.2)

Continuous variables	Mean (SD)
Age	25.9 (7.1)
Number of persons depending on worker's wage	4.0 (1.47)

Abbreviation: SD, standard deviation.

Mean HCC was 4.4 pg/mg (SD = 2.1 pg/mg, min–max 0.23–13.79 pg/mg) and mean ln(HCC) was 1.35 pg/mg (SD 0.53 pg/mg). Table 1 shows workplace stressors and resources as reported by the subsample of hair providers analyzed for this paper. Major stressors were having to keep one's job to support children/spouse or other relatives financially (96% and 61% agreement, respectively). An average of four people depended on a workers' wage. Only about a third of workers could call on someone for financial support if they lost their job.

Results of the association analysis of workplace stressors and resources with HCC can be found in Table 2. Most effect estimates were close to 0 and non‐significant. The sole stressor that was significant in fully adjusted models was “have to keep job to support children or spouse financially” (*β* = 0.28, 95% CI 0.02–0.55) indicating higher cortisol concentrations for individuals reporting that they have to support their spouse or children.

**TABLE 2:** Associations of HCC with workplace stressors and resources among *n* = 576 ready‐made garment workers.

	Model 1^a^			Model 2^b^			Model 3^c^		
Indicator	*β*	95% CI	*p*‐value	*β*	95% CI	*p*‐value	*β*	95% CI	*p*‐value
Job‐related stressors and resources									
Bullied by colleagues	.07	−0.05 to 0.18	.24	0.08	−0.04 to 0.19	.18	0.10	−0.01 to 0.22	.08
Bullied by supervisor	−.04	−0.16 to 0.08	.53	−0.03	−0.15 to 0.09	.59	−0.02	−0.14 to 0.10	.79
Trust in information from management	.02	−0.09 to 0.13	.72	−0.01	−0.19 to 0.10	.84	−0.03	−0.14 to 0.08	.62
Management trusts the employees (perceived)	−.11	−0.27 to 0.05	.18	−0.12	−0.27 to 0.04	.15	−0.14	−0.30 to 0.02	.09
Supervisors do not care about workers' problems	−.09	−0.17 to 0.00	.05	−0.08	−0.16 to 0.01	.07	−0.08	−0.16 to 0.01	.08
Supervisors take decisions free of personal bias	−.04	−0.14 to 0.05	.39	−0.03	−0.12 to 0.07	.55	−0.04	−0.13 to 0.60	.46
Support from colleagues	.01	−0.09 to 0.12	.81	−0.00	−0.10 to 0.10	.97	−0.01	−0.12 to 0.09	0.78
Support from supervisor	−0.03	−0.14 to 0.08	.62	−0.06	−0.17 to 0.05	.31	−0.08	‐ 0.19 to 0.03	.16
Family life has disturbed job	−.00	−0.12 to 0.11	.96	−0.00	−0.12 to 0.11	.98	−0.00	−0.12 to 0.11	.98
Problems in family due to job	.02	−0.11 to 0.15	.75	0.01	−0.11 to 0.14	.86	0.00	−0.12 to 0.13	.95
Financial support of children or spouse	.26	−0.00 to 0.53	.05	**0.30** *	**0.04–0.57** *	**.03** *	**0.28** *	**0.02–0.55** *	**.04** *
Financial support of other relatives	.04	−0.05 to 0.13	.39	0.03	−0.06 to 0.12	.54	0.02	−0.07 to 0.11	.68
Financial support available	**.14** **	**0.04–0.23** **	**.00** **	**0.09** *	**0.00–0.19** *	**.04** *	0.09	−0.00 to 0.18	.05
Any savings	−0.03	−0.13 to 0.08	.63	−0.03	−0.14 to 0.07	.55	−0.02	−0.12 to 0.08	.73
Amount of savings									
Less than 1 monthly salary	Ref.	Ref.	Ref.	Ref.	Ref.	Ref.	Ref.	Ref.	Ref.
1–2 monthly salaries	−.07	−0.22 to 0.08	.35	−0.05	−0.20 to 0.10	.53	−0.05	−0.20 to 0.10	.48
3 or more monthly salaries	−.02	−0.17 to 0.12	.75	−0.00	−0.15 to 0.14	.99	0.00	−0.14 to 0.15	.99
Any debt	−.06	−0.15 to 0.03	.19	−0.01	−0.10 to 0.08	.86	−0.02	−0.11 to 0.07	.67
Amount of debt									
Less than 1 monthly salary	Ref.	Ref.	Ref.	Ref.	Ref.	Ref.	Ref.	Ref.	Ref.
1–2 monthly salaries	−.07	−0.21 to 0.07	.32	−0.05	−0.18 to 0.09	.50	−0.06	−0.19 to 0.08	.43
3 or more monthly salaries	−.07	−0.18 to 0.04	.19	−0.02	−0.14 to 0.09	.67	−0.02	−0.13 to 0.09	.73
Number of persons depending on wage	.01	−0.03 to 0.03	.76	0.03	−0.01 to 0.06	.10	0.02	−0.01 to 0.05	.14

Abbreviation: HCC, hair cortisol concentrations.

^a^ Unadjusted for age, sex.

^b^ Adjusted for age, sex.

^c^ Adjusted for age, sex, poor self‐reported health, cardiovascular disease, diabetes, education.

* *p*‐value significant at α ≤ .05

** *p*‐value significant at α ≤ .01.

## 4 DISCUSSION

### 4.1 Discussion of findings

The aim of the present study was to investigate possible associations of workplace stressors and resources with HCC among RMG workers in Bangladesh. For nearly all investigated stressors and resources, we did not find meaningful associations. Having to support one's spouse or children financially was the sole stressor significantly associated with increased HCC. It is conceivable that individuals who have financial responsibilities may feel obliged to family members, resulting in increased stress levels. The importance of providing financial support to family members becomes apparent as 96% of participants agreed to support children/spouse financially, 61% supported other relatives, and an average of four people depended on a worker's salary. Well‐defined obligations within the kinship system (e.g., women expected to financially support not only children and their spouse but also their in‐laws) also likely lead to stress. Furthermore, it is likely that financial obligations weighed even more heavily on RMG workers in early 2021 than usually: The COVID‐19 pandemic and the far‐reaching lockdowns that prevailed in Bangladesh from March 2020 on had affected the entire economy of Bangladesh dramatically. The situation for informal sector workers, to which spouses and relatives of RMG workers usually belong, was particularly challenging. RMG workers were among the first to regain their jobs and might have therefore felt additional pressure to keep their jobs to support their unemployed relatives.

The item “have to keep job to support children or spouse financially” only applied to workers with children/spouse and almost all of respective workers agreed (96%). Therefore, the observed association between elevated HCC and having to support children or spouse financially cannot be disentangled from having children or a spouse. Neither having savings/debt nor suffering from work–family conflict were significantly associated with HCC in our study which suggests that there may be more at stake than mere financial or time conflicts leading to stress among RMG workers with children/spouse. From anthropological studies, we know of female workers suffering from a sense of responsibility and guilt toward their children whom they often leave behind in their villages,^8^ which may explain elevated HCC and which surpasses the concept of WFC captured in our study. In conclusion, both actual financial obligations exacerbated in the wake of the COVID‐19 pandemic (having to keep the job for financial support), but also emotional factors may explain the observed elevated HCC, both of which are related to having children and/or a spouse. In light of multiple testing, however, we cannot rule out this finding was random.

Both, this study and our previous study overall did not display meaningful associations of workplace stressors with elevated HCC (in both studies that each investigated different stressors only one stressor among a broad range of stressors was significant^4^). Compared to the previous study, the present study (i) followed a more rigorous sampling approach (i.e., random sampling of workers from various factories versus convenience sampling of workers from a single factory), (ii) included a much larger number of garment workers (576 vs. 175) and provided thus higher statistical power, and (iii) analyzed novel work‐related stressors identified in literature (e.g., workplace bullying and support, vertical trust between management and employees, work–family conflict, financial obligations).

According to a scoping review, evidence on the link between work‐related stressors and HCC remains inconsistent in international cross‐sectional and longitudinal research.^9^ The authors discuss, among others, small sample sizes and selection bias in recruiting participants as possible explanations for inconsistent findings. According to a meta‐analysis, human cortisol concentrations may even be lower in chronically stressed populations (so‐called “stress‐related hypocortisolism”).^10^ The authors present several explanations, for example, that chronic stress both increases and decreases cortisol concentrations but at different time points (e.g., cortisol increase at first exposure with a steady adaption over time). They also hypothesize how the exact nature of threat and its controllability may also influence cortisol secretion. These findings are in keeping with a Chinese study among employees in the petroleum and petrochemical sector that found elevated HCC levels in workers who experienced an increase of occupational stress in comparison with workers whose occupational stress remained constant (irrespective of the initial stress level).^11^

With respect to the remaining stressors/resources analyzed in our study, a possible explanation for the lack of evidence of meaningful associations may also be that hair samples collected in our study were 2 cm in length thereby referring to the past 2 months, whereas no reference period was given when inquiring after stressors/resources. We cannot rule out that a potential mismatch of reference periods between stressors/resources and HCC measurements contributed to our nil findings.

The mean HCC in our sample was rather low. To the best of our knowledge, no studies have yet investigated HCC among a sample in Bangladesh except for our own previous work that found even lower concentrations.^4^ That previous study also examined links between work stressors and HCC and additionally adjusted for smoking. We did not include smoking as confounder in the present analysis as various studies did not find associations between smoking and HCC.^12^ However, we ran post‐hoc analysis adjusting for smoking, but obtained similar results as in our primary analysis. One study analyzing samples from India found comparatively low levels of HCC^13^ while other international studies among various populations report higher concentrations.^14^ Environmental and lifestyle factors (e.g., traffic and air pollution,^15^ hair treatment^12^) may also influence HCC.

### 4.2 Strengths and limitations

Our study has several strengths. Firstly, the random sampling approach of participants likely reduced selection bias and increased the representativeness of the drawn sample for the overall RMG worker population. We surveyed a large number of workers from various factories and therefore with likely various working conditions and conducted interviews outside of working hours to additionally reduce selection bias and a possible healthy worker effect. Nevertheless, some limitations apply. We used a self‐devised interview with unknown validity and reliability. However, the items were derived based on prior qualitative research and the instrument was piloted which likely increased face validity and comprehensibility of items. Only about half of interview participants provided hair samples so that we cannot rule out selection bias considering the provision of hair. Post‐hoc comparison of hair providers and non‐providers showed that the former suffered from poorer self‐reported health than the latter. No differences were observed with respect to sex, age, or marital status. No causal relationships can be derived due to the study's cross‐sectional design.

## 5 CONCLUSIONS

Among all investigated workplace stressors and resources of RMG workers in Bangladesh, the sole workplace stressor significantly associated with increased levels of HCC was having to keep one's job to support children or spouse financially. This stressors can however barely be disentangled from the fact that one has children/a spouse. Future studies may focus on different markers (e.g., blood samples) and perform longitudinal research to better examine causality.

## AUTHOR CONTRIBUTIONS

Annegret Dreher was involved in the conceptualization of the study, the formal analysis, and visualization of the data as well as writing the original manuscript draft. Rita Yusuf was involved in the conceptualization of the study, the project administration and supervision and in reviewing/editing of the manuscript. Hasan Ashraf was involved in the conceptualization of the study and reviewing/editing of the manuscript. Syed A. K. Shifat Ahmed was involved in the conceptualization of the study, the training and supervision of the survey staff, data curation and reviewing/editing of the manuscript. Wei Gao was involved in interpretation of the data and reviewing/editing of the manuscript. Christian Strümpell was involved in conceptualization of the study, funding acquisition and reviewing/editing of the final manuscript. Adrian Loerbroks was responsible for the conceptualization of the study, funding acquisition, project administration, supervision, and reviewing/editing of the final manuscript.

## CONFLICT OF INTEREST STATEMENT

Authors declare no Conflict of Interests for this article.

## Data Availability

The data that support the findings of this study are available from the corresponding author upon reasonable request.

## APPENDIX 1

**TABLE A1:** List of variables and their respective coding for linear regression analysis.

Variable name	Variable coding for regression analysis
Dependent variable	
Hair cortisol concentration	Continuous (pg/mg)
Independent variables	
Bullied by colleagues	1 = Yes 0 = No
Bullied by supervisor	1 = Yes 0 = No
Trust in information from management	1 = Yes 0 = No
Management trusts the employees (perceived)	1 = Yes 0 = No
Supervisors do not care about workers' problems	1 = Yes 0 = No
Supervisors take decisions free of personal bias	1 = Yes 0 = No
Support from colleagues	1 = Yes 0 = No
Support from supervisor	1 = Yes 0 = No
Family life has disturbed job	1 = Yes 0 = No
Problems in family due to job	1 = Yes 0 = No
Financial support of children or spouse	1 = Yes 0 = No
Financial support of other relatives	1 = Yes 0 = No
Financial support available	1 = Yes 0 = No
Any savings	1 = Yes 0 = No
Amount of savings	0 = Less than 1 monthly salary 1 = 1–2 monthly salaries 2 = 3 or more monthly salaries
Any debt	1 = Yes 0 = No
Amount of debt	0 = Less than 1 monthly salary 1 = 1–2 monthly salaries 2 = 3 or more monthly salaries
Number of persons depending on wage	Continuous (number)
